# Evaluation of clinically available renal biomarkers in critically ill adults: a prospective multicenter observational study

**DOI:** 10.1186/s13054-017-1626-0

**Published:** 2017-03-07

**Authors:** Yujun Deng, Ruibin Chi, Shenglong Chen, Heng Ye, Jie Yuan, Lin Wang, Yiling Zhai, Lu Gao, Danqing Zhang, Linhui Hu, Bo Lv, Yi Long, Cheng Sun, Xiaobing Yang, Xia Zou, Chunbo Chen

**Affiliations:** 1 Department of Critical Care Medicine, Guangdong General Hospital, Guangdong Academy of Medical Sciences, 106 Zhongshan Er Road, Guangzhou, 510080 Guangdong Province People’s Republic of China; 2 Department of Critical Care Medicine, Xiaolan Hospital of Southern Medical University, 65 Jucheng Road, Zhongshan, 528415 Guangdong People’s Republic of China; 3 Department of Critical Care Medicine, Guangzhou Nansha Central Hospital, 105 Fengzhe Road, Nansha, 511400 Guangdong People’s Republic of China; 4 National Clinical Research Center for Kidney Disease, Nanfang Hospital, Southern Medical University, 1838 Guangzhou Road, Guangzhou, 510515 Guangdong People’s Republic of China; 5 School of Public Health, Sun Yat-sen University, 74 Zhongshan Er Road, Guangzhou, 510080 Guangdong Province People’s Republic of China

**Keywords:** Acute kidney injury, Renal biomarker, Serum cystatin C, *N*-acetyl-β-d-glucosaminidase, Urinary albumin/creatinine ratio, Intensive care unit

## Abstract

**Background:**

Although serum cystatin C (sCysC), urinary *N*-acetyl-β-d-glucosaminidase (uNAG), and urinary albumin/creatinine ratio (uACR) are clinically available, their optimal combination for acute kidney injury (AKI) detection and prognosis prediction remains unclear. We aimed to assess the discriminative abilities of these biomarkers and their possible combinations for AKI detection and intensive care unit (ICU) mortality prediction in critically ill adults.

**Methods:**

A multicenter, prospective observational study was conducted in mixed medical-surgical ICUs at three tertiary care hospitals. One thousand eighty-four adult critically ill patients admitted to the ICUs were studied. We assessed the use of individual biomarkers (sCysC, uNAG, and uACR) measured at ICU admission and their combinations with regard to AKI detection and prognosis prediction.

**Results:**

AUC-ROCs for sCysC, uNAG, and uACR were calculated for total AKI (0.738, 0.650, and 0.683, respectively), severe AKI (0.839, 0.706, and 0.771, respectively), and ICU mortality (0.727, 0.793, and 0.777, respectively). The panel of sCysC plus uNAG detected total and severe AKI with significantly higher accuracy than either individual biomarkers or the other two panels (uNAG plus uACR or sCysC plus uACR). For detecting total AKI, severe AKI, and ICU mortality at ICU admission, this panel yielded AUC-ROCs of 0.756, 0.863, and 0.811, respectively; positive predictive values of 0.71, 0.31, and 0.17, respectively; and negative predictive values of 0.81, 0.97, and 0.98, respectively. Moreover, this panel significantly contributed to the accuracy of the clinical models for AKI detection and ICU mortality prediction, as measured by the AUC-ROC, continuous net reclassification index, and incremental discrimination improvement index. The comparable performance of this panel was further confirmed with bootstrap internal validation.

**Conclusions:**

The combination of a functional marker (sCysC) and a tubular damage marker (uNAG) revealed significantly superior discriminative performance for AKI detection and yielded additional prognostic information on ICU mortality.

**Electronic supplementary material:**

The online version of this article (doi:10.1186/s13054-017-1626-0) contains supplementary material, which is available to authorized users.

## Background

Acute kidney injury (AKI) is increasingly prevalent [1, 2], and it is associated with adverse outcomes [3–5]. Delayed diagnosis of AKI impedes timely intervention [6], and thus early identification of AKI is critical. However, AKI is a heterogeneous syndrome that represents a complex multifactorial clinical condition. It is speculated that a single biomarker will be insufficiently sensitive and specific across the full spectrum of AKI, and combinations of biomarkers with different characteristics (e.g., combination of a functional biomarker and a tubular damage biomarker) may prove more accurate in a complex clinical setting [7–9]. However, the optimal combination of biomarkers for clinical use remains a challenge, especially in a heterogeneous population.

Serum cystatin C (sCysC) and urinary albumin/creatinine ratio (uACR) are glomerular filtration biomarkers for AKI, whereas urinary *N*-acetyl-β-d-glucosaminidase (uNAG) is a tubular damage biomarker [8, 10]. These biomarkers are clinically available in European, North American, and Asian centers. CysC, which is produced in all nucleated cells, is freely filtered in glomeruli and completely absorbed, catabolized by proximal tubular cells, and not secreted into the urine by the tubule [8]. Hence, CysC is considered a better marker of glomerular filtration rate than serum creatinine (sCr) [8] and can predict AKI and adverse outcomes [10, 11]. *N*-acetyl-β-d-glucosaminidase (NAG), which originates from proximal and distal tubular cells and nonrenal cells, is released into urine following tubular damage [8, 10]. Because its large size precludes its glomerular filtration, uNAG is a quite sensitive marker that reflects renal tubule damage [12, 13]. It manifested well as an early damage biomarker of AKI and could predict poor outcomes [12, 14]. Albumin, a small amount of which can pass through the filtration barrier, is reabsorbed by the proximal tubule normally [10]. Because increased urinary albumin reflects the increased permeability of the basal membrane of glomerular injury [15] and indicates glomerular structure and function change, it is considered a useful diagnostic tool for renal disease [10, 16], including AKI [17, 18]. Nevertheless, the availability of high-quality evidence validating the performance of these biomarkers and their combinations for AKI detection and prognosis prediction in heterogeneous cohorts is insufficient. Therefore, we conducted a large, prospective, multicenter observational study in adult general intensive care units (ICUs) to assess the performance of these individual biomarkers and their possible combinations at ICU admission with respect to AKI detection and prognosis prediction.

## Methods

### Study design and participants

The present prospective observational study was conducted in the general ICUs of three tertiary care hospitals in China. All consecutive patients between October 2014 and February 2016 were eligible for enrollment. The exclusion criteria included age under 18 years, refusal of consent, nephrectomy, end-stage renal disease (ESRD), renal transplant, preexisting dialysis before ICU admission, or missing admission data. The outcome variables were the detection of AKI within 1 week of ICU enrollment and ICU mortality. The study protocol met Strengthening the Reporting of Observational Studies in Epidemiology [19] and Standards for Reporting Diagnostic Accuracy [20] criteria. The study protocol was approved by the local institutional review board.

### Sample and data collection

Blood and urine samples were collected simultaneously within 1 h after ICU admission. All samples collected from the participating hospitals were shipped by commercial cold chain transportation and analyzed batched after collection and storage. All samples were measured at the central laboratory of the Guangdong General Hospital using a standard protocol within 24 h after collection. Baseline clinical characteristics were prospectively collected. sCysC, uNAG, and uACR were measured once at ICU admission. sCr was measured at ICU admission and thereafter at least once daily as a part of routine clinical care during ICU hospitalization. The hourly urine output from enrollment to ICU discharge was also recorded. The following clinical variables were evaluated: age, sex, body mass index (BMI), preexisting clinical conditions, sepsis, admission type, baseline sCr, baseline estimated glomerular filtration rate (eGFR), sCr at ICU admission, Acute Physiology and Chronic Health Evaluation (APACHE) II score, length of ICU stay, length of hospital stay, renal replacement therapy (RRT) during ICU stay, ICU mortality, and in-hospital mortality. The baseline eGFR was estimated by the simplified Modification of Diet in Renal Disease formula [21].

### Definitions

AKI was diagnosed according to the Kidney Disease: Improving Global Outcomes (KDIGO) criteria for AKI within 1 week after ICU admission [22] as any of the following: increase in sCr by ≥0.3 mg/dl (≥26.5 μmol/L) within 48 h, or increase in sCr to ≥1.5 times baseline within 1 week, or urine output <0.5 ml/kg/h for 6 h. AKI is staged according to the following KDIGO criteria. Stage 1 is an increase of sCr to 1.5–1.9 times baseline, or ≥0.3 mg/dl (≥26.5 μmol/L) increase of sCr, or urine output <0.5 ml/kg/h for 6–12 h. Stage 2 is an increase of sCr to 2.0–2.9 times from baseline or urine output <0.5 ml/kg/h for ≥12 h. Stage 3 is a an increase of sCr to three times baseline, or ≥4.0 mg/dl (≥353.6 μmol/L) increase of sCr, or initiation of RRT, or urine output <0.3 ml/kg/h for ≥24 h or anuria for ≥12 h.

A baseline creatinine was determined using the following rules ranked in descending order of preference as previously described [23]: (1) the most recent pre-ICU value between 30 and 365 days before ICU admission (*n* = 141); (2) a stable pre-ICU value >365 days for patients aged <40 years (stable defined as within 15% of the lowest ICU measurement) before ICU admission (*n* = 3); (3) pre-ICU value >365 days before ICU admission and less than the initial sCr at ICU admission (*n* = 35); (4) a pre-ICU value (between 3 and 39 days before ICU admission) less than or equal to the initial sCr on admission to ICU and not distinctly in AKI (*n* = 515); or (5) the lowest sCr upon initial admission to ICU (*n* = 113), the last ICU value (*n* = 156), or the minimum value at follow-up up to 365 days (*n* = 121). Severe AKI was defined as KDIGO stage 2 or stage 3 within 1 week after ICU admission. Established AKI indicated the diagnosis of AKI at ICU admission. Later-onset AKI was defined as no AKI diagnosis at ICU admission but reaching the KDIGO criteria within 1 week after admission. Progressive AKI was defined as worsening of AKI stage in patients with established AKI (from stage 1 to either stage 2 or stage 3, or from stage 2 to stage 3). The diagnosis of sepsis was defined according to the American College of Chest Physicians/Society of Critical Care Medicine Consensus Conference Committee guidelines [24].

### Biomarker assays

sCysC and sCr, urinary creatinine, uNAG, and albumin levels were measured using the UniCel DxC 800 Synchron system (Beckman Coulter, Brea, CA, USA) according to the manufacturer’s instructions. The coefficients of interassay and intraassay variation in sCysC were <5% and ≤10%, respectively. The coefficients of interassay and intraassay variation for uNAG were both ≤10%. The interassay and intraassay coefficients of variation for urinary albumin were both <10%. Both the values of uNAG and albumin were normalized to urinary creatinine concentration. The personnel measuring all the biomarkers were blinded to each patient’s clinical characteristics. Because the stability of sCysC and uNAG has already been demonstrated [25–27], urinary albumin will not degrade significantly with short-term storage [28, 29]. Preanalysis about the influence of cooling or freezing of samples was not executed.

### Statistical analysis

The SPSS version 13.0 (SPSS, Chicago, IL, USA), R version 3.3.1 (R Foundation for Statistical Computing, Vienna, Austria), and MedCalc version 12.5.0 (MedCalc Software, Ostend, Belgium) software programs were used for statistical analysis. Continuous variables were presented as median (IQR). Categorical variables were expressed as number (percent). The nonnormally distributed continuous variables were compared by Wilcoxon rank-sum test or Kruskal-Wallis test for one-way analysis of variance. If the Kruskal-Wallis test showed statistical significance, a post hoc Steel-Dwass test was subsequently conducted. To compare the categorical variables, the chi-square test or Fisher’s exact test was used. If the three biomarkers displayed nonnormal distributions, a nonparametric Spearman’s test was then used to assess the correlation.

The ROC curves with their AUCs were calculated. The comparison of AUCs between the groups was conducted with the method developed by DeLong et al. [30], and the optimal combination (with highest AUCs) was included in the subsequent analyses. The sensitivity, specificity, positive and negative predictive values (PPV and NPV, respectively), and positive and negative likelihood ratios ([−] LR and [+] LR, respectively) of the biomarkers were also calculated. The optimal cutoff values for AKI detection and ICU mortality were defined for individual biomarkers and their combinations using Youden’s index [31].

The performance of the optimal panel combined with the reference clinical model was assessed by AUC, integrated discrimination improvement (IDI) index, and continuous net reclassification improvement (cNRI) index, as described previously [32, 33]. We conducted univariate and multivariate logistic regression to construct the clinical models. The clinical variables with *P* < 0.10 in univariate analysis were included in multivariate analysis. A stepwise method was used for variable selection.

The performance of the optimal panel for AKI detection and ICU mortality prediction was internally validated by a bootstrap method with 1000 replications [34]. All the tests were two-tailed, and *P* < 0.05 was considered statistically significant.

## Results

### Patient characteristics

Of the 1162 consecutive adult patients screened for inclusion in the study, 78 (6.7%) were excluded for the following reasons: refusal to consent (*n* = 15), nephrectomy (*n* = 3), kidney transplant (*n* = 3), missing admission data (*n* = 34), ESRD, or undergoing RRT before ICU admission (*n* = 23). Thus, 1084 (93.3%) patients were enrolled in the analysis. AKI occurred in 326 patients (30.1%).

Patient characteristics are shown in Table 1. Compared with the non-AKI patients, the patients with AKI were older and had a higher rate of preexisting clinical conditions, such as diabetes mellitus (DM), hypertension, chronic kidney disease (CKD), chronic liver disease, stroke, chronic obstructive pulmonary disease (COPD), coronary artery disease (CAD), heart failure (HF), and cancer. Worse renal function was observed in patients with AKI. Patients with AKI had a higher concentration of sCr and higher APACHE II scores at ICU admission, and they had adverse outcomes. Three hundred twenty-eight cases (30.3%) were complicated with sepsis at ICU admission. The incidence of AKI (54.6%) was more frequent in patients with sepsis.

**Table 1:** Baseline characteristics and outcomes

Characteristics	Non-AKI (*n* = 758)	AKI (*n* = 326)	*P* value
Demographic variables			
Age, years	52.0 (41.0–62.0)	62.1 (47.4–73.0)	<0.001
Male sex, *n* (%)	393 (51.8)	197 (60.4)	0.009
BMI, kg/m^2^	22.2 (21.6–23.1)	22.4 (21.8–23.3)	0.324
Preexisting clinical conditions			
Hypertension, *n* (%)	117 (15.4)	115 (35.3)	<0.001
DM, *n* (%)	42 (5.5)	51 (15.6)	<0.001
CKD, *n* (%)	16 (2.1)	44 (13.5)	<0.001
Chronic liver disease, *n* (%)	10 (1.3)	19 (5.8)	<0.001
Stroke, *n* (%)	80 (10.6)	73 (22.4)	<0.001
COPD, *n* (%)	16 (2.1)	14 (4.3)	0.044
CAD, *n* (%)	20 (2.6)	32 (9.8)	<0.001
HF, *n* (%)	13 (1.7)	30 (9.2)	<0.001
Cancer, *n* (%)	79 (10.4)	52 (16.0)	0.01
Thyroid disease, *n* (%)	23 (3.0)	14 (4.3)	0.295
Sepsis, *n* (%)	149 (19.7)	179 (54.9)	<0.001
Admission type, *n* (%)			<0.001
Elective surgical, *n* (%)	555 (73.2)	110 (33.7)	
Emergency surgical, *n* (%)	88 (11.6)	68 (20.9)	
Medical, *n* (%)	115 (15.2)	148 (45.4)	
Baseline serum creatinine, mg/dl	0.69 (0.58–0.83)	0.74 (0.57–0.95)	0.018
Baseline eGFR, ml/minute/1.73 m^2^	110.20 (94.40–133.60)	105.60 (77.15–141.70)	0.033
Serum creatinine at admission, mg/dl	0.77 (0.64–0.92)	1.07 (0.82–1.45)	<0.001
APACHE II score	10 (8–14)	17 (11–26)	<0.001
UP, ml/kg/h	1.99 (1.48–2.62)	1.76 (1.08–2.46)	<0.001
Outcomes			
Length of ICU stay, days	3 (2–4)	5 (3–10)	<0.001
Length of hospital stay, days	12 (8–16)	14 (9–23)	<0.001
RRT during ICU stay, *n* (%)	4 (0.5)	20 (6.1)	<0.001
ICU mortality, *n* (%)	20 (2.6)	46 (14.1)	<0.001
In-hospital mortality, *n* (%)	28 (3.7)	51 (15.6)	<0.001

*Abbreviations: AKI* Acute kidney injury; *BMI* Body mass index; *DM* Diabetes mellitus; *CAD*, Coronary artery disease; *COPD*, Chronic obstructive pulmonary disease; *HF*, Heart failure; *CKD*, Chronic kidney disease, defined as baseline estimated glomerular filtration rate <60 ml/minute/1.73 m^2^; *eGFR*, Estimated glomerular filtration rate; *APACHE*, Acute Physiology and Chronic Health Evaluation; *UP*, Urine production first 24 h after admission; *ICU*, Intensive care unit; *RRT*, Renal replacement therapy

The nonnormally distributed continuous variables are expressed as median (25th percentile to 75th percentile IQR). Categorical variables are expressed as *n* (%)

### AKI detection by biomarkers measured at ICU admission

**Fig. 1:**
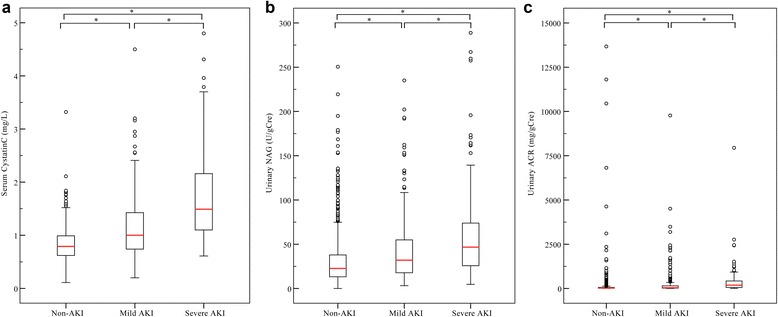
Admission concentrations of the three biomarkers, stratified by AKI severity. **a** sCysC. **b** uNAG. **c** uACR. Concentrations of the three biomarkers are shown in each AKI severity category (non-AKI [*n* = 758], mild AKI [stage 1; *n* = 224], severe AKI [stage 2 and stage 3; *n* = 102]). * *P* < 0.05. *AKI* Acute kidney injury, *Cre* Creatinine, *sCysC* Serum cystatin C, *uNAG* Urinary *N*-acetyl-β-d-glucosaminidase, *uACR* Urinary albumin/creatinine ratio

Of 326 patients with AKI, 102 had severe AKI. The ROC curve analysis revealed that the three studied biomarkers detected AKI with statistical significance (Table 2). The AUC-ROC values of sCysC for detecting total and severe AKI were higher than those of uNAG or uACR. The three biomarkers appeared to be increased along with the severity of AKI (Fig. 1). Interestingly, the biomarker concentrations were significantly correlated with one another (Additional file 1: Table S1), with the strongest correlation being between the two urinary biomarkers.

**Table 2:** Three biomarkers for total AKI and severe AKI detection

Biomarkers	Non-AKI^a^ (*n* = 758)	Total AKI^a^ (*n* = 326)	AUC-ROC^b^ (95% CI)
		Severe AKI^a^ (*n* = 102)	
sCysC (mg/L)	0.79 (0.62–0.98)	1.13 (0.80–1.57)	0.738 (0.703–0.772)^c,d^
		1.49 (1.10–2.16)	0.839 (0.798–0.880)^c,d^
uNAG (U/g Cre)	22.63 (13.21–37.93)	35.28 (20.36–66.53)	0.650 (0.614–0.686)^e^
		46.73 (25.64–75.07)	0.706 (0.651–0.761)^d,e^
uACR (mg/g Cre)	23.90 (11.54–60.30)	73.65 (22.41–264.90)	0.683 (0.648–0.718)^e^
		187.82 (54.75–428.71)	0.771 (0.726–0.817)^c,e^

*Abbreviations: AKI* Acute kidney injury, sCysC, Serum cystatin C, *uNAG* Urinary *N*-acetyl-β-d-glucosaminidase, *Cre* Creatinine concentration, *uACR* Urinary albumin/creatinine ratio

^a^ The nonnormally distributed continuous variables are expressed as median (25th percentile to 75th percentile [interquartile range])

^b^ Values are presented as AUC-ROC (95% confidence interval)

^c^ *P* < 0.05 vs. uNAG

^d^ *P* < 0.05 vs. uACR

^e^ *P* < 0.05 vs. sCysC

**Fig. 2:**
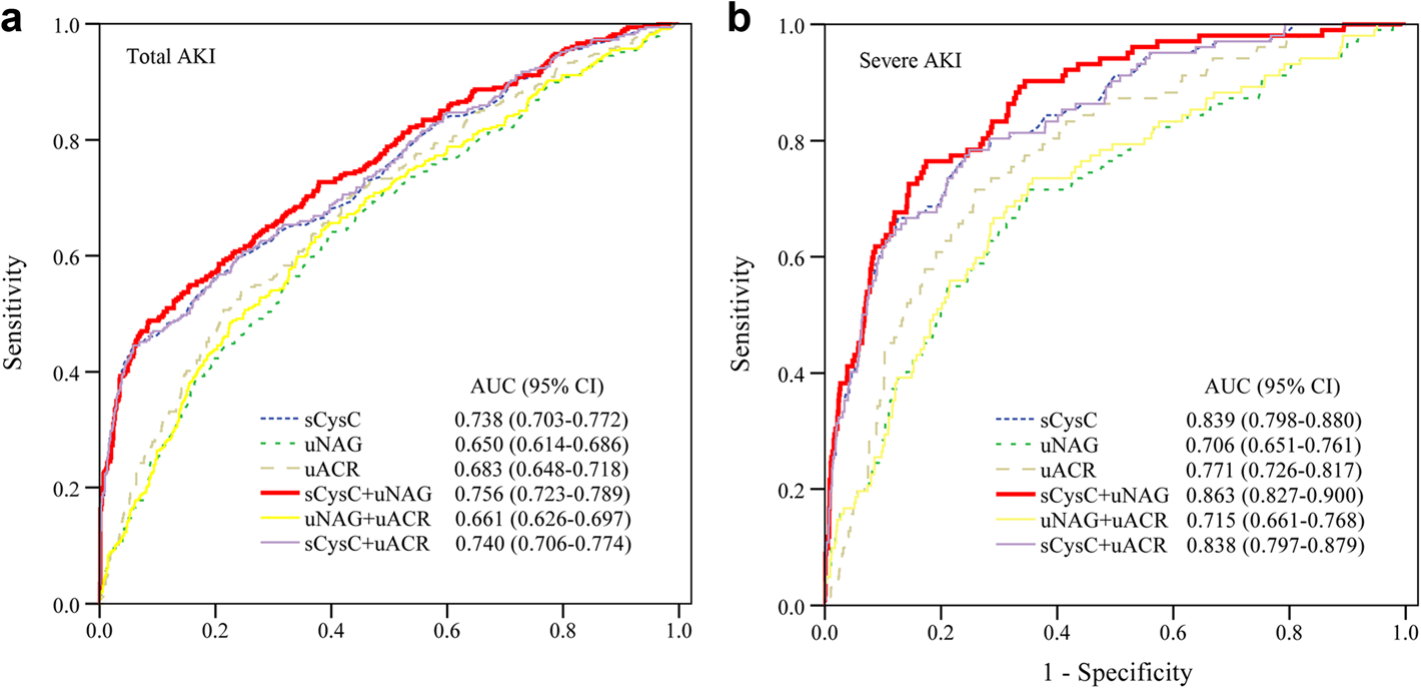
ROC analysis of three biomarkers and their combinations for AKI detection. Among 1084 adult critically ill patients, 326 (30.07%) were diagnosed with AKI (**a** total AKI). Of 326 patients with AKI, 102 patients were diagnosed with severe AKI (**b** severe AKI). *AKI* Acute kidney injury, *ICU* Intensive care unit, *sCysC* Serum cystatin C, *uNAG* Urinary *N*-acetyl-β-d-glucosaminidase, *uACR* Urinary albumin/creatinine ratio

To improve the performance of these biomarkers in AKI detection, we developed three possible panels consisting of these biomarkers (sCysC plus uNAG, uNAG plus uACR, and sCysC plus uACR) (Table 3). sCysC identified total AKI and severe AKI with high specificity but limited sensitivity. uNAG detected total AKI and severe AKI with relatively high sensitivity but low specificity. uACR detected total AKI and severe AKI with a sensitivity of 54% and 72% and a specificity of 76% and 74%, respectively. The AUC-ROCs for total AKI and severe AKI demonstrated better performance by the panel of sCysC plus uNAG than by either the individual biomarkers or the other two panels (Table 3 and Fig. 2). Thus, the panel of sCysC plus uNAG was selected for the subsequent analyses. Of 326 patients with AKI, 120 patients were diagnosed with later-onset AKI, whereas the other 206 patients were diagnosed with established AKI (Additional file 2: Table S2). Among the 206 patients with established AKI, 29 had progressive AKI. The three biomarkers demonstrated poor to moderate AUC-ROC values for predicting later-onset AKI and progressive AKI. The panel of sCysC plus uNAG had the highest AUC-ROC value for the prediction of later-onset AKI (Additional file 3: Table S3). The AUC-ROC values of this panel for later-onset AKI and severe later-onset AKI were 0.667 and 0.837, respectively. However, this panel’s AUC-ROC value for progressive AKI was 0.756, which was not superior to that of sCysC alone (Additional file 4: Table S4).

**Table 3:** Detective characteristics of the three biomarkers and their combinations for total acute kidney injury and severe acute kidney injury

Logistic regression model	AUC-ROC^a^	Cutoff^b^	Sensitivity	Specificity	(+) LR	(−) LR	PPV	NPV
Total AKI (*n* = 326)								
Univariate models								
sCysC	0.738 (0.703–0.772)	1.26 mg/L	0.44	0.95	7.92	0.59	0.77	0.80
uNAG	0.650 (0.614–0.686)	27.14 U/g Cre	0.64	0.60	1.59	0.60	0.41	0.80
uACR	0.683 (0.648–0.718)	61.14 mg/g Cre	0.54	0.76	2.20	0.61	0.49	0.79
Multivariate models								
sCysC + uNAG	0.756 (0.723–0.789)^c^	0.43^d^	0.49	0.91	5.69	0.56	0.71	0.81
uNAG + uACR	0.661 (0.626–0.697)^e^	0.27^d^	0.64	0.62	1.68	0.58	0.42	0.80
sCysC + uACR	0.740 (0.706–0.774)^f^	0.45^d^	0.45	0.94	7.66	0.59	0.77	0.80
Severe AKI (*n* = 102)								
Univariate models								
sCysC	0.839 (0.798–0.880)	1.25 mg/L	0.67	0.87	5.28	0.38	0.35	0.96
uNAG	0.706 (0.651–0.761)	32.80 U/g Cre	0.72	0.65	2.03	0.44	0.17	0.96
uACR	0.771 (0.726–0.817)	71.97 mg/g Cre	0.72	0.74	2.77	0.38	0.22	0.96
Multivariate models								
sCysC + uNAG	0.863 (0.827–0.900)^c^	0.09^d^	0.76	0.83	4.39	0.28	0.31	0.97
uNAG + uACR	0.715 (0.661–0.768)^g^	0.08^d^	0.74	0.64	2.06	0.41	0.18	0.96
sCysC + uACR	0.838 (0.797–0.879)^f^	0.08^d^	0.78	0.75	3.16	0.29	0.25	0.97

*Abbreviations: (+) LR* Positive likelihood ratio, *(−) LR* negative likelihood ratio, *PPV* Positive predictive value, *NPV* Negative predictive value, *sCysC* Serum cystatin C, *uNAG* Urinary *N*-acetyl-β-d-glucosaminidase, *Cre* Creatinine concentration, *uACR* Urinary albumin/creatinine ratio

^a^ Values are presented as AUC-ROC (95% CI)

^b^ Ideal cutoff value according to Youden’s index

^c^ *P* < 0.05 vs. sCysC, uNAG, uACR, uNAG + uACR, and sCysC + uACR

^d^ Cutoff points of the biomarker panels were the predicted probabilities generated from the multiple logistic regression model

^e^ *P* < 0.05 vs. sCysC, uNAG, sCysC + uACR, and sCysC + uNAG

^f^ *P* < 0.05 vs. uNAG, uACR, uNAG + uACR, and sCysC + uNAG

^g^ *P* < 0.05 vs. sCysC, uNAG, uACR, sCysC + uACR, and sCysC + uNAG

### Biomarkers in septic AKI

We further evaluated the performance of the three biomarkers in patients with sepsis (Additional file 5: Table S5). sCysC, uNAG, and uACR were able to discriminate AKI in patients with sepsis. Moreover, sCysC had significant diagnostic superiority over the other biomarkers for detecting septic AKI and severe septic AKI. The AUC-ROC values of sCysC for detecting septic AKI and severe septic AKI were 0.784 and 0.812, respectively. In contrast, uNAG showed poor AUC-ROC values for detecting septic AKI and severe septic AKI. However, none of the biomarker combinations demonstrated adequate predictive characteristics when compared with sCysC alone for detecting septic AKI (Additional file 6: Table S6). It is noteworthy that the median value of uNAG in patients with sepsis who did not develop AKI was higher than that in patients with AKI of entire cohort (Table 2 and Additional file 5: Table S5). Among these patients with sepsis without AKI, 1 exhibited a positive urine culture with *Staphylococcus aureus*, 1 patient had a positive result for renal abscess with *S. aureus*, 1 had a positive result for urine culture with *Enterococcus faecium*, 1 had a positive urine culture with *Candida tropicalis*, 1 had a positive urine culture with *Candida glabrata*, and 15 showed significantly high white blood cell counts in the urine sediment without a positive culture under antibiotic treatment. These 20 patients’ median uNAG value on admission was 37.93 U/g Cre (24.52–51.62), with the highest value being 110.70 U/g Cre and the lowest value being 10.79 U/g Cre.

### Mortality and RRT prediction by biomarkers measured at ICU admission

**Fig. 3:**
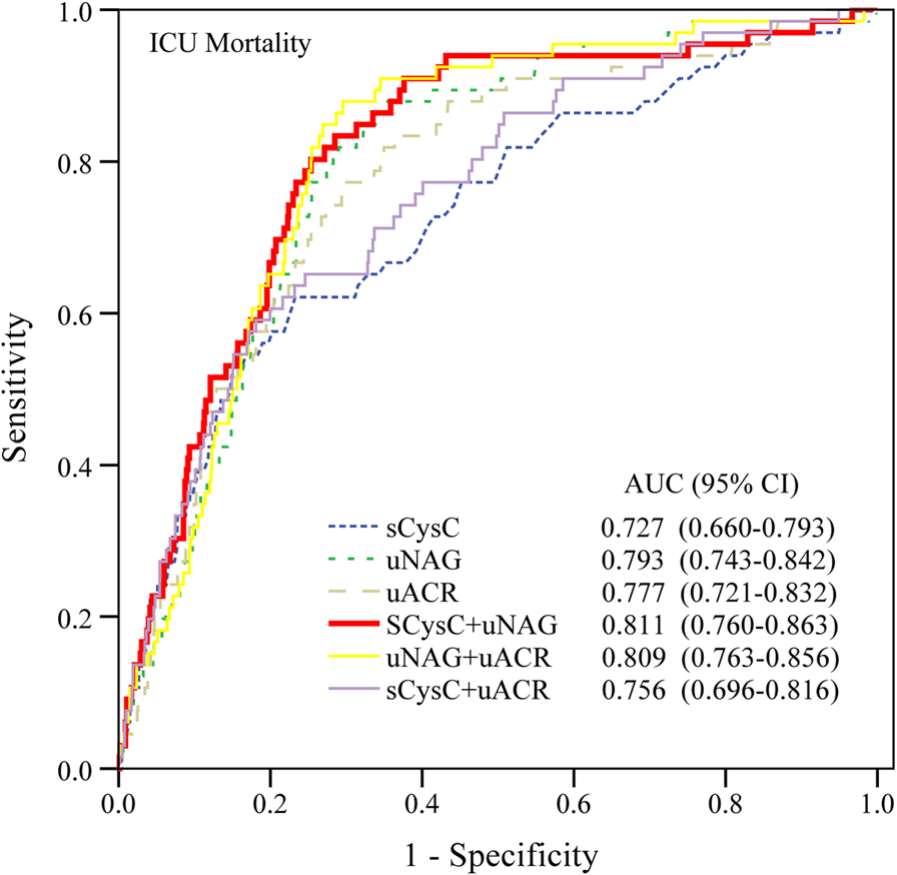
ROC analysis of the three biomarkers and their combinations for ICU mortality. Among 1084 adult critically ill patients, 66 patients died in the ICU. *ICU* Intensive care unit, *sCysC* Serum cystatin C, *uNAG* Urinary *N*-acetyl-β-d-glucosaminidase, *uACR* Urinary albumin/creatinine ratio

In the entire cohort, no significant differences between sCysC, uNAG, uACR for predicting RRT during ICU stay, ICU mortality, or in-hospital mortality were detected (Additional file 7: Table S7). The predictive abilities of biomarker combinations with respect to ICU mortality were assessed (Table 4 and Fig. 3). The panel of sCysC plus uNAG had the highest AUC of those of the individual biomarkers or the other two panels. The AUC-ROC of sCysC improved to 0.811 with the addition of uNAG. Thus, the panel of sCysC plus uNAG for ICU mortality prediction was selected for the subsequent analyses.

**Table 4:** Predictive characteristics of admission biomarkers and their combinations for intensive care unit mortality

Logistic regression model	AUC-ROC^a^	Cutoff^b^	Sensitivity	Specificity	(+) LR	(−) LR	PPV	NPV
Univariate models								
sCysC	0.727 (0.660–0.793)	1.12 mg/L	0.62	0.77	2.67	0.49	0.15	0.97
uNAG	0.793 (0.743–0.842)	37.75 U/g Cre	0.82	0.71	2.83	0.26	0.16	0.98
uACR	0.777 (0.721–0.832)	63.66 mg/g Cre	0.77	0.70	2.61	0.32	0.15	0.98
Multivariate models								
sCysC+ uNAG	0.811 (0.760–0.863)^c,d^	0.05^e^	0.80	0.75	3.17	0.26	0.17	0.98
uNAG + uACR	0.809 (0.763–0.856)^f^	0.05^e^	0.88	0.70	2.97	0.17	0.16	0.99
sCysC + uACR	0.756 (0.696–0.816)	0.06^e^	0.59	0.82	3.27	0.50	0.18	0.97

*Abbreviations: (+) LR* Positive likelihood ratio, *(−) LR* Negative likelihood ratio, *PPV* Positive predictive value, *NPV* Negative predictive value, *sCysC* Serum cystatin C, *uNAG* Urinary *N*-acetyl-β-d-glucosaminidase, *Cre* Creatinine concentration, *uACR* Urinary albumin/creatinine ratio

^a^ Values are presented as AUC-ROC (95% CI). Among 1084 adult critically ill patients, 66 patients died in the intensive care unit

^b^ Ideal cutoff value according to Youden’s index

^c^ *P* < 0.05 vs. sCysC

^d^ *P* < 0.05 vs. sCysC + uACR

^e^ Cutoff points of the biomarker panels were the predicted probabilities generated from the multiple logistic regression model

The performance of sCysC plus uNAG was further confirmed by bootstrap internal validation, in which the average AUC-ROC values for detecting total AKI, severe AKI, and ICU mortality were 0.757 (95% CI 0.724–0.789), 0.863 (95% CI 0.827–0.899), and 0.812 (95% CI 0.758–0.865), respectively.

### Relative contribution of the panel of sCysC plus uNAG to the clinical model for AKI detection and ICU mortality prediction

To determine the added contribution of this panel to the clinical models for AKI detection and ICU mortality, logistic regression analysis was performed. Potential available variables at ICU admission for AKI detection included sex; age; BMI; sCr at admission; baseline eGFR; sepsis; admission type; and preexisting clinical conditions, including DM, hypertension, chronic liver disease, stroke, COPD, CAD, HF, cancer, and thyroid disease. The potential predictors for ICU mortality prediction included the above-mentioned variables and APACHE II score. The model for total AKI detection included sex, sCr at admission, sepsis, admission type, and chronic liver disease. The model for severe AKI detection contained sCr at admission, sepsis, and admission type. The model for ICU mortality prediction was composed of APACHE II score and admission type. After the models were constructed (Additional file 8: Table S8), the panel of sCysC plus uNAG was added to the above-described models to assess the improvement in the discriminative ability. Adding this panel increased the total AKI and ICU mortality model’s AUC-ROC values significantly. However, the ROC curve analysis demonstrated that the addition of this panel did not yield statistically significant improvement from the model for detecting severe AKI. Moreover, adding this panel to the clinical models for detecting AKI or ICU mortality improved their predictive abilities, as measured by the cNRI and IDI indices.

## Discussion

The main finding of the present multicenter study was that the panel of sCysC plus uNAG showed superior discriminative performance in AKI detection when compared with either the individual biomarkers or the other two panels, and it also provided critical prognostic information. To our knowledge, the present study demonstrates for the first time that a panel of sCysC plus uNAG yields greater predictive abilities for AKI in an adult general ICU cohort.

Several potential serum and urine biomarkers of kidney injury have been identified, such as neutrophil gelatinase-associated lipocalin (NGAL) [35, 36], kidney injury molecule 1 [37], interleukin 18 [38], NAG [14], CysC [39], urinary albumin [40], tissue inhibitor of metalloproteinase 2, and insulin-like growth factor-binding protein 7 [41]. Among them, sCysC, uNAG, and uACR are clinically available in China and other countries. However, most studies so far have been focused on their individual abilities to detect AKI in ICU patients and have yielded inconsistent performance [42–44], and the predictive abilities of their combinations in adult general ICU patients have not yet been determined.

Because AKI is a heterogeneous syndrome, a single biomarker is not sensitive or specific enough to reflect the multiple pathophysiologies of AKI [12]. Promisingly, the Acute Dialysis Quality Initiative (ADQI) working group recommended that a reformulation of the diagnostic approach for AKI include not only the markers of function but also markers of kidney damage, without the need for changes in kidney function [9]. It is reasonable that a combination of functional and tubular damage biomarkers for AKI, which reflects different underlying pathological processes in the generation of AKI, may be superior to individual biomarkers alone. Researchers in several studies have investigated different combinations of biomarkers for predicting AKI, and they reported improved predictive performance for the various combinations they used [42, 45–47]. Our findings are consistent with those studies. In the present cohort, the combination of sCysC and uNAG at ICU admission was an adequate predictor of AKI compared with either the individual biomarkers or the other two panels.

CysC is a glomerular function marker and can predict AKI and adverse outcomes [10, 11]. However, a wide range of its predictive accuracy for AKI and severe outcomes has been found [26, 43, 48]. In our study, sCysC demonstrated significantly higher AUC-ROC values for detecting AKI than those of uNAG or uACR. Moreover, the specificity of sCysC for AKI detection or ICU mortality prediction was much greater than that of uNAG or uACR, whereas its sensitivity was limited. Urinary albumin is another functional biomarker for renal function [10]. The use of uACR as a biomarker for AKI was shown in recent studies [15, 40]. However, the ability of uACR in predicting AKI also varied across investigations [15, 40, 43, 44]. In our cohort, uACR demonstrated poor to moderate AUC-ROC values for AKI detection. uNAG manifested well as an early damage biomarker of AKI and also could predict poor outcomes [12, 14]. Although NAG is sensitive in reflecting renal tubule damage, its specificity for AKI is limited [12, 43]. Furthermore, its predictive abilities for AKI and outcomes also differed across various published studies [10, 43]. In the present study, uNAG showed poor to moderate AUC-ROC values for AKI detection. Moreover, its sensitivity for AKI detection and ICU mortality prediction was higher than its specificity.

In this study, sCysC served as a functional biomarker with high specificity for AKI detection and prognosis prediction, and uNAG served as a tubular damage biomarker with relatively higher sensitivity than its specificity. The combination of sCysC and uNAG yielded greater diagnostic performance in detecting AKI and predicting adverse outcome. This combination’s superiority may be attributed to the fact that the combination, consisting of a functional biomarker with high specificity and a tubular damage biomarker with high sensitivity, reflects different damaging mechanisms of the nephron. Furthermore, the specimens for this panel included serum and urine samples. The urinary biomarkers may potentially be more sensitive to intrinsic histological damage, and serum levels of biomarkers may be more sensitive to changes in clearance [43]. The present study indicated that a combination of different characteristics and various sources of specimens (serum and urine) may be a reasonable strategy to provide a better biomarker panel for AKI diagnosis and prognosis in complicated clinical settings. These findings are consistent with those of a previous study in which investigators reported that a panel consisting of a functional biomarker (plasma CysC) plus tubular damage biomarker (urinary NGAL) improved the predictive ability for discrete characteristics of AKI in cardiac surgery patients [47]. On one hand, our findings add to the evidence that a combination of biomarkers with different sensitivity and specificity improves diagnostic performance [45]. On the other hand, the panel of uNAG plus uACR and the panel of sCysC plus uACR in this cohort failed to improve diagnostic performance substantially. One potential explanation is that the former panel included the same source of specimens, and the latter panel included two functional biomarkers that are clustered together, indicating glomerular function, and share the same mechanism [49]*.* The consequence of our investigation may be another proof and appending of more evidence to such a study domain. However, we found poor performance of uNAG and lack of superiority of the combination (sCysC plus uNAG) versus sCysC for detecting septic AKI. One possible explanation is that uNAG level increased in patients with urinary tract infection, regardless of AKI complication. In addition, significantly elevated concentration of uNAG may be associated with sepsis because uNAG in the patients with sepsis complicated with AKI showed the highest values.

Our study has limitations. First, we measured these three biomarkers only once at ICU admission. As ADQI cannot recommend a serial testing schedule [9], it is not practical and cost-effective for collecting and measuring a series of samples at frequent time points. We speculate that our conclusions are not debilitated by this limitation. Second, only 58 patients with CKD were enrolled, and thus we could not stratify our cohort according to the baseline eGFR. Therefore, future studies should be conducted in this subgroup. Last, the internal and external validity of this study should be noted because the timing, etiology, and amount of renal impact cannot be exactly known, and patients with established AKI or undergoing surgery dominated in the present heterogeneous cohort, which may blur the accuracy for AKI detection.

## Conclusions

The present study shows that the combination of a functional marker (sCysC) and a tubular damage marker (uNAG) at ICU admission had significantly better discriminative performance for AKI detection than either the individual biomarkers or the other two panels, and that combining this panel with a clinical model added significant value for AKI detection. Moreover, this panel also significantly contributed to the accuracy of the clinical model for ICU mortality prediction. This study was conducted in general adult ICUs with a heterogeneous cohort. Thus, our findings could have significant clinical implications for actual heterogeneous ICU patients at risk for AKI.

## Key messages

- The clinically available renal biomarkers (sCysC, uNAG, and uACR) can detect AKI and ICU mortality in critically ill patients.
- The panel of sCysC plus uNAG at ICU admission showed superior discriminative performance in AKI detection when compared with either the individual biomarkers or the other two panels, and also provided additional prognostic information on ICU mortality.

## Additional files

Additional file 1:
**Table S1.** Correlations among three biomarkers at ICU admission. The correlation of biomarkers with one another at ICU admission. (DOCX 14 kb)
Additional file 2:
**Table S2.** AUC-ROC of biomarkers for established AKI, later-onset AKI and progressive AKI. AUC-ROC values for detection of established AKI, late-onset AKI or progressive AKI. (DOCX 14 kb)
Additional file 3:
**Table S3.** Predictive characteristics of admission biomarkers and their combinations for later-onset AKI. Values of AUC-ROC, cutoff, sensitivity, specificity, (+) LR, (−) LR, PPV, and NPV for these biomarkers and their combinations for predicting later-onset AKI. (DOCX 18 kb)
Additional file 4:
**Table S4.** Predictive characteristics of admission biomarkers and their combinations for progressive AKI. Values of AUC-ROC, cutoff, sensitivity, specificity, (+) LR, (−) LR, PPV, and NPV for these biomarkers and their combinations for predicting progressive AKI. (DOCX 16 kb)
Additional file 5:
**Table S5.** Three biomarkers for AKI detection in patients with sepsis. Values of AUC-ROC and concentration of three biomarkers for detecting septic AKI or severe septic AKI. (DOCX 16 kb)
Additional file 6:
**Table S6.** Predictive characteristics of admission biomarkers and their combinations for AKI in sepsis Patients. Values of AUC-ROC, cutoff, sensitivity, specificity, (+) LR, (−) LR, PPV, and NPV for these biomarkers and their combinations for detecting septic AKI or severe septic AKI. (DOCX 19 kb)
Additional file 7
**Table S7.** AUC-ROC for renal replacement therapy and mortality prediction by biomarkers and APACHE II score. AUC-ROC values of three biomarkers and APACHE II score for prediction of renal replacement therapy and mortality. (DOCX 14 kb)
Additional file 8:
**Table S8.** AUC-ROC, NRI and IDI when biomarkers were added to the clinical models. Values of AUC-ROC, continuous NRI, and IDI when the combination (sCysC and uNAG) was added to the clinical models for detection total AKI, severe AKI, or ICU mortality. (DOCX 16 kb)

## Abbreviations

(+) LR: Positive likelihood ratio
(−) LR: Negative likelihood ratio
ACR: Albumin/creatinine ratio
ADQI: Acute Dialysis Quality Initiative
AKI: Acute kidney injury
APACHE: Acute Physiology and Chronic Health Evaluation
BMI: Body mass index
CAD: Coronary artery disease
CKD: Chronic kidney disease, defined as baseline estimated glomerular filtration rate <60 ml/minute/1.73 m^2^
cNRI: Continuous net reclassification improvement index
COPD: Chronic obstructive pulmonary disease
DM: Diabetes mellitus
eGFR: Estimated glomerular filtration rate
ESRD: End-stage renal disease
HF: Heart failure
ICU: Intensive care unit
IDI: Integrated discrimination improvement index
KDIGO: Kidney Disease: Improving Global Outcomes
NAG: *N*-acetyl-β-d-glucosaminidase
NGAL: Neutrophil gelatinase-associated lipocalin
NPV: Negative predictive value
PPV: Positive predictive value
RRT: Renal replacement therapy
sCr: Serum creatinine
sCysC: Serum cystatin C
uACR: Urinary albumin/creatinine ratio
uNAG: Urinary *N*-acetyl-β-d-glucosaminidase
