# Supplementary material for: Evaluation of clinically available renal biomarkers in critically ill adults: a prospective multicenter observational study
Source: Crit Care. 2017 Mar 7;21:46. doi: 10.1186/s13054-017-1626-0 (PMC5339963; doi:10.1186/s13054-017-1626-0)
Supplement: Additional file 1: — Table S1. Correlations among three biomarkers at ICU admission. The correlation of biomarkers with one another at ICU admission. (DOCX 14 kb) [file 13054_2017_1626_MOESM1_ESM.docx]

**Table S1. Correlations among three biomarkers at ICU admission**

| **Spearman’s rho** | **sCysC (mg/L)** | **uACR (mg/g Cre)** |
| --- | --- | --- |
| uNAG (U/g Cre) | 0.23**^*^** | 0.38**^*^** |
| uACR(mg/g Cre) | 0.24**^*^** |  |

All correlations **^*^***P*<0.01. SCysC, serum Cystatin C; uNAG, urinary N-acetyl-ß-D-glucosaminidase; Cre, creatinine concentration; uACR, urinary albumin/creatinine ratio.
