# Supplementary material for: Evaluation of clinically available renal biomarkers in critically ill adults: a prospective multicenter observational study
Source: Crit Care. 2017 Mar 7;21:46. doi: 10.1186/s13054-017-1626-0 (PMC5339963; doi:10.1186/s13054-017-1626-0)
Supplement: Additional file 2: — Table S2. AUC-ROC of biomarkers for established AKI, later-onset AKI and progressive AKI. AUC-ROC values for detection of established AKI, late-onset AKI or progressive AKI. (DOCX 14 kb) [file 13054_2017_1626_MOESM2_ESM.docx]

**Table S2. AUC-ROC of biomarkers for established AKI, later-onset AKI and progressive AKI^a^**

| **Biomarkers** | **Established AKI (n=206)** | **Later-onset AKI (n=120)** | **Progressive AKI (n=29)** |
| --- | --- | --- | --- |
| sCysC (mg/L) | 0.791 (0.753-0.829)**^#$^** | 0.621 (0.565-0.676) | 0.720 (0.622-0.818) |
| uNAG (U/g Cre) | 0.638 (0.594-0.681)**^*$^** | 0.640 (0.587-0.694) | 0.644 (0.534-0.754) |
| uACR(mg/g Cre) | 0.686 (0.645-0.727)**^*#^** | 0.644 (0.590-0.698) | 0.715 (0.613-0.817) |

**^a^**Values are presented as AUC-ROC (95% confidence interval). AUC-ROC, area under the receiver operating characteristic curve; AKI, acute kidney injury; sCysC, serum Cystatin C; uNAG, urinary N-acetyl-ß-D-glucosaminidase; Cre, creatinine concentration; uACR, urinary albumin/creatinine ratio.

**^*^***P*<0.05. vs. sCysC; **^#^***P*<0.05 vs. uNAG; **^$^***P*<0.05. vs. uACR.
