# Supplementary material for: Evaluation of clinically available renal biomarkers in critically ill adults: a prospective multicenter observational study
Source: Crit Care. 2017 Mar 7;21:46. doi: 10.1186/s13054-017-1626-0 (PMC5339963; doi:10.1186/s13054-017-1626-0)
Supplement: Additional file 3: — Table S3. Predictive characteristics of admission biomarkers and their combinations for later-onset AKI. Values of AUC-ROC, cutoff, sensitivity, specificity, (+) LR, (−) LR, PPV, and NPV for these biomarkers and their combinations for predicting later-onset AKI. (DOCX 18 kb) [file 13054_2017_1626_MOESM3_ESM.docx]

**Table S3. Predictive characteristics of admission biomarkers and their combinations for later-onset AKI**

| **Logistic regression**  **model** | **AUC-ROC^a^** | **Cut-off^b^** | **Sensitivity** | **Specificity** | **(+) LR** | **(-) LR** | **PPV** | **NPV** |
| --- | --- | --- | --- | --- | --- | --- | --- | --- |
| **Total later-onset AKI (n=120)** |  |  |  |  |  |  |  |  |
| **Univariate models** |  |  |  |  |  |  |  |  |
| sCysC | 0.621 (0.565-0.676) | 1.27 mg/L | 0.24 | 0.95 | 4.47 | 0.80 | 0.41 | 0.89 |
| uNAG | 0.640 (0.587-0.694) | 28.12 U/g Cre | 0.63 | 0.62 | 1.65 | 0.60 | 0.21 | 0.91 |
| uACR | 0.644 (0.590-0.698) | 62.07 mg/g Cre | 0.48 | 0.76 | 1.99 | 0.68 | 0.24 | 0.90 |
| **Multivariate models** |  |  |  |  |  |  |  |  |
| sCysC + uNAG | 0.667 (0.615-0.719)**^¶^** | 0.18**^c^** | 0.38 | 0.85 | 2.64 | 0.72 | 0.30 | 0.90 |
| uNAG + uACR | 0.652 (0.598-0.705) | 0.12**^c^** | 0.73 | 0.54 | 1.57 | 0.51 | 0.20 | 0.93 |
| uACR + sCysC | 0.631 (0.577-0.686) | 0.16**^c^** | 0.40 | 0.79 | 1.90 | 0.76 | 0.23 | 0.89 |
| **Severe** **later-onset AKI (n=24)** |  |  |  |  |  |  |  |  |
| **Univariate models** |  |  |  |  |  |  |  |  |
| sCysC | 0.723 (0.640-0.805) | 0.78 mg/L | 0.92 | 0.48 | 1.76 | 0.17 | 0.05 | 1.00 |
| uNAG | 0.767 (0.685-0.850) | 34.48 U/g Cre | 0.79 | 0.69 | 2.57 | 0.30 | 0.07 | 0.99 |
| uACR | 0.746 (0.661-0.831) | 65.34 mg/g Cre | 0.63 | 0.74 | 2.43 | 0.51 | 0.06 | 0.99 |
| **Multivariate models** |  |  |  |  |  |  |  |  |
| sCysC + uNAG | 0.837 (0.783-0.890)**^§^** | 0.02**^c^** | 0.96 | 0.62 | 2.53 | 0.07 | 0.07 | 1.00 |
| uNAG + uACR | 0.770 (0.688-0.851) | 0.02**^c^** | 0.79 | 0.69 | 2.59 | 0.30 | 0.07 | 0.99 |
| uACR + sCysC | 0.729 (0.646-0.812) | 0.02**^c^** | 0.92 | 0.48 | 1.76 | 0.17 | 0.05 | 1.00 |

**^a^**Values are presented as AUC-ROC (95% confidence interval); **^b^**Ideal cut-off value according to Youden’s index; ^c^Cut-off points of the biomarker panels were the predicted probability generated from the multiple logistic regression model; AUC-ROC, area under the receiver operating characteristic curve; AKI, acute kidney injury; (+) LR, positive likelihood ratio; (-) LR, negative likelihood ratio; PPV, positive predictive value; NPV, negative predictive value; sCysC, serum Cystatin C; uNAG, urinary N-acetyl-ß-D-glucosaminidase; Cre, creatinine concentration; uACR, urinary albumin/creatinine ratio. **^¶^***P*<0.05 vs. sCysC and sCysC + uACR; **^§^***P*<0.05 vs. sCysC, uACR, and sCysC + uACR.
