# Supplementary material for: Evaluation of clinically available renal biomarkers in critically ill adults: a prospective multicenter observational study
Source: Crit Care. 2017 Mar 7;21:46. doi: 10.1186/s13054-017-1626-0 (PMC5339963; doi:10.1186/s13054-017-1626-0)
Supplement: Additional file 4: — Table S4. Predictive characteristics of admission biomarkers and their combinations for progressive AKI. Values of AUC-ROC, cutoff, sensitivity, specificity, (+) LR, (−) LR, PPV, and NPV for these biomarkers and their combinations for predicting progressive AKI. (DOCX 16 kb) [file 13054_2017_1626_MOESM4_ESM.docx]

**Table S4. Predictive characteristics of admission biomarkers and their combinations for progressive AKI**

| **Logistic regression**  **model** | **AUC-ROC^a^** | **Cut-off ^b^** | **Sensitivity** | **Specificity** | **(+) LR** | **(-) LR** | **PPV** | **NPV** |
| --- | --- | --- | --- | --- | --- | --- | --- | --- |
| **Univariate models** |  |  |  |  |  |  |  |  |
| sCysC | 0.720 (0.622-0.818) | 1.39 mg/L | 0.76 | 0.61 | 1.95 | 0.40 | 0.26 | 0.93 |
| uNAG | 0.644 (0.534-0.754) | 32.89 U/g Cre | 0.79 | 0.50 | 1.60 | 0.41 | 0.23 | 0.93 |
| uACR | 0.715 (0.613-0.817) | 193.21 mg/g Cre | 0.62 | 0.76 | 2.60 | 0.50 | 0.32 | 0.92 |
| **Multivariate models** |  |  |  |  |  |  |  |  |
| sCysC + uNAG | 0.756 (0.668-0.844)**^§^** | 0.14**^c^** | 0.79 | 0.67 | 2.43 | 0.31 | 0.31 | 0.95 |
| uNAG +uACR | 0.678 (0.576-0.779) | 0.14**^c^** | 0.72 | 0.65 | 2.06 | 0.43 | 0.27 | 0.93 |
| uACR+ sCysC | 0.726 (0.628-0.824) | 0.14**^c^** | 0.76 | 0.63 | 2.04 | 0.38 | 0.27 | 0.94 |

**^a^**Values are presented as AUC-ROC (95% confidence interval); **^b^**Ideal cut-off value according to Youden’s index; ^c^Cut-off points of the biomarker panels were the predicted probability generated from the multiple logistic regression model. Of 206 patients, 29 patients were diagnosed as progressive AKI. AKI, acute kidney injury; AUC-ROC, area under the receiver operating characteristic curve; (+) LR, positive likelihood ratio; (-) LR, negative likelihood ratio; PPV, positive predictive value; NPV, negative predictive value; sCysC, serum Cystatin C; uNAG, urinary N-acetyl-ß-D-glucosaminidase; Cre, creatinine concentration; uACR, urinary albumin/creatinine ratio. **^§^***P*<0.05 vs. uNAG.
