# Supplementary material for: Evaluation of clinically available renal biomarkers in critically ill adults: a prospective multicenter observational study
Source: Crit Care. 2017 Mar 7;21:46. doi: 10.1186/s13054-017-1626-0 (PMC5339963; doi:10.1186/s13054-017-1626-0)
Supplement: Additional file 5: — Table S5. Three biomarkers for AKI detection in patients with sepsis. Values of AUC-ROC and concentration of three biomarkers for detecting septic AKI or severe septic AKI. (DOCX 16 kb) [file 13054_2017_1626_MOESM5_ESM.docx]

**Table S5. Three biomarkers for AKI detection in patients with sepsis**

| **Biomarkers** | **Sepsis^a^**  **(n=328)** | **Non-AKI sepsis^a^**  **(n=149)** | **Septic AKI^a^ (n = 179)**  **Severe septic AKI^a^ (n = 74 )** | **AUC-ROC^b^**  **(95% CI)** |
| --- | --- | --- | --- | --- |
| sCysC (mg/L) | 1.06 (0.78-1.47) | 0.86 (0.68-1.07) | 1.39 (0.97-1.81)  1.53 (1.25-2.31) | 0.784 (0.735-0.833)^#$^  0.812 (0.760-0.864)^#$^ |
| uNAG (U/g Cre) | 39.43 (21.57-67.35) | 36.00 (20.11-61.10) | 43.24 (24.23-73.56)  47.30 (25.64-75.07) | 0.569 (0.507-0.631)^*$^  0.584 (0.509-0.658)^*$^ |
| uACR (mg/g Cre) | 97.85 (26.53-293.83) | 52.84 (17.85-188.35) | 142.26 (42.79-405.48)  238.34 (87.36-445.31) | 0.647 (0.588-0.707)^*#^  0.681 (0.616-0.745)^*#^ |

**^a^**The non-normally distributed continuous variables are expressed as median (25th percentile to 75th percentile [interquartile range]); **^b^**Values are presented as AUC-ROC (95% confidence interval). AUC-ROC, area under the receiver operating characteristic curve; CI, Confidence Interval; AKI, acute kidney injury; sCysC, serum Cystatin C; uNAG, urinary N-acetyl-ß-D-glucosaminidase; Cre, creatinine concentration; uACR, urinary albumin/creatinine ratio. **^*^***P*<0.05 vs. sCysC; **^#^***P*<0.05 vs. uNAG; **^$^***P*<0.05 vs. uACR.
