# Supplementary material for: Evaluation of clinically available renal biomarkers in critically ill adults: a prospective multicenter observational study
Source: Crit Care. 2017 Mar 7;21:46. doi: 10.1186/s13054-017-1626-0 (PMC5339963; doi:10.1186/s13054-017-1626-0)
Supplement: Additional file 6: — Table S6. Predictive characteristics of admission biomarkers and their combinations for AKI in sepsis Patients. Values of AUC-ROC, cutoff, sensitivity, specificity, (+) LR, (−) LR, PPV, and NPV for these biomarkers and their combinations for detecting septic AKI or severe septic AKI. (DOCX 19 kb) [file 13054_2017_1626_MOESM6_ESM.docx]

**Table S6. Predictive characteristics of admission biomarkers and their combinations for AKI in sepsis patients**

| **Logistic regression**  **model** | **AUC-ROC^a^** | **Cut-off^b^** | **Sensitivity** | **Specificity** | **(+) LR** | **(-) LR** | **PPV** | **NPV** |
| --- | --- | --- | --- | --- | --- | --- | --- | --- |
| **Septic AKI (n=179)** |  |  |  |  |  |  |  |  |
| **Univariate models** |  |  |  |  |  |  |  |  |
| sCysC | 0.784 (0.735-0.833) | 1.22 mg/L | 0.60 | 0.88 | 4.95 | 0.46 | 0.86 | 0.65 |
| uNAG | 0.569 (0.507-0.631) | 30.15 U/g Cre | 0.68 | 0.46 | 1.26 | 0.70 | 0.60 | 0.54 |
| uACR | 0.647 (0.588-0.707) | 71.79 mg/g Cre | 0.68 | 0.58 | 1.62 | 0.55 | 0.66 | 0.60 |
| **Multivariate models** |  |  |  |  |  |  |  |  |
| sCysC + uNAG | 0.785 (0.736-0.834)**^§^** | 0.61**^c^** | 0.60 | 0.88 | 4.95 | 0.46 | 0.87 | 0.65 |
| uNAG +uACR | 0.588 (0.527-0.650)**^¶^** | 0.51**^c^** | 0.75 | 0.44 | 1.33 | 0.58 | 0.62 | 0.59 |
| sCysC + uACR | 0.784 (0.735-0.833)**^§^** | 0.60**^c^** | 0.61 | 0.88 | 5.04 | 0.44 | 0.86 | 0.65 |
| **Septic severe AKI (n=74)** |  |  |  |  |  |  |  |  |
| **Univariate models** |  |  |  |  |  |  |  |  |
| sCysC | 0.812 (0.760-0.864) | 1.25 mg/L | 0.76 | 0.75 | 3.00 | 0.33 | 0.47 | 0.91 |
| uNAG | 0.584 (0.509-0.658) | 32.80 U/g Cre | 0.73 | 0.45 | 1.33 | 0.60 | 0.28 | 0.85 |
| uACR | 0.681 (0.616-0.745) | 54.63 mg/g Cre | 0.85 | 0.47 | 1.59 | 0.32 | 0.32 | 0.92 |
| **Multivariate models** |  |  |  |  |  |  |  |  |
| sCysC + uNAG | 0.813 (0.761-0.865)**^§^** | 0.20**^c^** | 0.76 | 0.76 | 3.10 | 0.32 | 0.48 | 0.91 |
| uNAG +uACR | 0.589 (0.516-0.662)**^ǁ^** | 0.20**^c^** | 0.74 | 0.45 | 1.36 | 0.57 | 0.28 | 0.86 |
| sCysC + uACR | 0.810 (0.758-0.862)**^§^** | 0.16**^c^** | 0.82 | 0.65 | 2.33 | 0.27 | 0.40 | 0.93 |

**^a^**Values are presented as AUC-ROC (95% confidence interval); **^b^**Ideal cut-off value according to Youden’s index; ^c^Cut-off points of the biomarker panels were the predicted probability generated from the multiple logistic regression model. Of 328 patients with sepsis, 179 patients were diagnosed as AKI according to the KEDIGO criteria, 74 patients were diagnosed as Septic severe AKI (severe AKI was defined as KDIGO stages 2 and 3). AKI, Acute kidney injury; AUC-ROC, area under the receiver operating characteristic curve; (+) LR, positive likelihood ratio; (-) LR, negative likelihood ratio; PPV, positive predictive value; NPV, negative predictive value; sCysC, serum Cystatin C; uNAG, urinary N-acetyl-ß-D-glucosaminidase; Cre, creatinine concentration; uACR, urinary albumin/creatinine ratio. **^§^***P*<0.05 vs. uNAG, uACR, and uNAG + uACR; **^¶^***P*<0.05 vs. sCysC, uNAG, sCysC + uACR, and sCysC + uNAG; **^ǁ^***P*<0.05 vs. sCysC, uACR, sCysC + uACR, and sCysC + uNAG.
