# Supplementary material for: Evaluation of clinically available renal biomarkers in critically ill adults: a prospective multicenter observational study
Source: Crit Care. 2017 Mar 7;21:46. doi: 10.1186/s13054-017-1626-0 (PMC5339963; doi:10.1186/s13054-017-1626-0)
Supplement: Additional file 7 — Table S7. AUC-ROC for renal replacement therapy and mortality prediction by biomarkers and APACHE II score. AUC-ROC values of three biomarkers and APACHE II score for prediction of renal replacement therapy and mortality. (DOCX 14 kb) [file 13054_2017_1626_MOESM7_ESM.docx]

**Table S7. AUC-ROC for renal replacement therapy and mortality prediction by biomarkers and APACHE II score^a^**

| **Biomarkers** | **RRT in ICU**  **(n=24)** | **Death in ICU**  **(n=66)** | **Death in hospital**  **(n=79)** |
| --- | --- | --- | --- |
| sCysC (mg/L) | 0.790 (0.695-0.885)**^*^** | 0.727 (0.660-0.793)**^*^** | 0.724 (0.663-0.784)**^*^** |
| uNAG (U/g Cre) | 0.798 (0.721-0.876)**^*^** | 0.793 (0.743-0.842)**^*^** | 0.760 (0.709-0.811)**^*^** |
| uACR (mg/g Cre) | 0.848 (0.792-0.904) | 0.777 (0.721-0.832)**^*^** | 0.757 (0.702-0.811)**^*^** |
| APACHE II | 0.891 (0.852-0.931) | 0.907 (0.881-0.932) | 0.891 (0.859-0.923) |

**^a^**Values are presented as AUC-ROC (95% confidence interval). AKI, acute kidney injury; AUC-ROC, area under the receiver operating characteristic curve; RRT, renal replacement therapy; sCysC, serum Cystatin C; uNAG, urinary N-acetyl-ß-D-glucosaminidase; Cre, creatinine concentration; uACR, urinary albumin/creatinine ratio; APACHE II, Acute Physiology and Chronic Health Evaluation score. **^*^***P*<0.05, vs. APACHE II score.
