# Supplementary material for: Evaluation of clinically available renal biomarkers in critically ill adults: a prospective multicenter observational study
Source: Crit Care. 2017 Mar 7;21:46. doi: 10.1186/s13054-017-1626-0 (PMC5339963; doi:10.1186/s13054-017-1626-0)
Supplement: Additional file 8: — Table S8. AUC-ROC, NRI and IDI when biomarkers were added to the clinical models. Values of AUC-ROC, continuous NRI, and IDI when the combination (sCysC and uNAG) was added to the clinical models for detection total AKI, severe AKI, or ICU mortality. (DOCX 16 kb) [file 13054_2017_1626_MOESM8_ESM.docx]

**Table S8. AUC-ROC, NRI and IDI when biomarkers were added to the clinical models**

| **Variables** | **AUC-ROC** | ***P*-Value^§^** | **IDI (95% CI)** | ***P*-Value** | **Continuous NRI (95% CI)** | ***P*-Value** |
| --- | --- | --- | --- | --- | --- | --- |
| **Total AKI** |  |  |  |  |  |  |
| Clinical model**^*^** | 0.821 (0.792-0.850) |  |  |  |  |  |
| +sCysC + uNAG | 0.836 (0.808-0.864) | 0.0051 | 0.022 (0.012-0.032) | <0.001 | 0.412 (0.285-0.538) | <0.001 |
| **Severe AKI** |  |  |  |  |  |  |
| Clinical model**^**^** | 0.908 (0.881-0.934) |  |  |  |  |  |
| + sCysC + uNAG | 0.918 (0.893-0.944) | 0.0885 | 0.031 (0.007-0.054) | 0.0107 | 0.517 (0.316-0.719) | <0.001 |
| **ICU mortality** |  |  |  |  |  |  |
| Clinical model**^#^** | 0.907 (0.884-0.930) |  |  |  |  |  |
| + sCysC + uNAG | 0.913 (0.890-0.935) | 0.0499 | 0.010 (-0.004-0.024) | 0.1477 | 0.414 (0.166-0.661) | 0.0010 |

AKI, acute kidney injury; AUC-ROC, area under the receiver operating characteristic curve; NRI, net reclassification improvement index; IDI, integrated discrimination improvement index; CI, Confidence Interval; sCysC, serum Cystatin C; uNAG, urinary N-acetyl-ß-D-glucosaminidase; uACR, urinary albumin/creatinine ratio. **^*^**The clinical model for detecting total AKI is composed of sex, admission serum creatinine, sepsis and admission type, chronic liver disease; **^**^**The clinical model for predicting severe AKI is composed of admission serum creatinine, sepsis and admission type; **^#^**The clinical model for predicting ICU mortality is composed of APACHE II score and admission type; **^§^**Biomarkers + clinical model vs. clinical model.
